# Learning from failure: how eliminating required attendance sparked the beginning of a medical school transformation

**DOI:** 10.1007/s40037-020-00615-y

**Published:** 2020-08-17

**Authors:** Sara Lamb, Candace Chow, Janet Lindsley, Adam Stevenson, Danielle Roussel, Kerri Shaffer, Wayne Samuelson

**Affiliations:** 1 Department of Internal Medicine and Pediatrics, University of Utah School of Medicine, 30 N. 1900 E., Salt Lake City, UT 84132 USA; 2 Department of Internal Medicine, University of Utah School of Medicine, 30 N. 1900 E., Salt Lake City, UT 84132 USA; 3 Department of Biochemistry, University of Utah School of Medicine, 30 N. 1900 E., Salt Lake City, UT 84132 USA; 4 Department of Pediatrics, University of Utah School of Medicine, 30 N. 1900 E., Salt Lake City, UT 84132 USA; 5 Department of Anesthesiology, University of Utah School of Medicine, 30 N. 1900 E., Salt Lake City, UT 84132 USA; 6 Dean’s Office, Office of Medical Education, Curriculum, University of Utah School of Medicine, 30 N. 1900 E., Salt Lake City, UT 84132 USA; 7 Department of Internal Medicine, University of Utah School of Medicine, 30 N. 1900 E., Salt Lake City, UT 84132 USA

**Keywords:** Attendance, Learning environment, Learner-teacher relationship

## Abstract

Concern about medical student attendance has been rising over the last decade. Thinking a required attendance policy would fix things, we instituted such a mandate in 2010 only to find that although students were present at lecture and other learning sessions they were disengaged. In addition, we experienced growing distrust between faculty and students and tensions between the Student Affairs and Curriculum offices. After five years, we dismantled the policy in favor of encouraged attendance. We discuss both positive and negative surprising consequences that followed this new approach to attendance which has reshaped our vision for the medical school learning experience. It has been transformative and has afforded us the opportunity to redefine our results in accord with the culture in which we aspire to live and work.

## The story

Research shows that the relationship between classroom attendance and academic performance in medical school is variable [1]. Some studies report a positive association between student attendance and the United States Medical Licensing Examination (USMLE) Step 1 scores [2] while others report a positive relationship between student attendance and course performance [3, 4]. Still others find no relationship between attendance and performance [5, 6]. Despite these mixed findings, student attendance (or lack thereof) is an important issue in the medical education community as evidenced by frequent list-serve queries and surveys on attendance policies [7]. The 2017 Association of American Medical Colleges (AAMC) Medical School Year Two Questionnaire All Schools Summary Report [7] reads: “Compared to previous classes, second-year medical students in 2017 were less likely to report attending in-person classes for pre-clerkship courses or lectures. Fewer than half (47.3%) reported having attended in-person pre-clerkship courses or lectures at their medical school ‘Most of the time’ (34.7%) or ‘Often’ (12.6%). This continues a decline observed in prior years.” Not surprisingly, we have struggled with this issue at our institution.

In 2010, the University of Utah School of Medicine (UUSOM) introduced a new curriculum. Scheduled class time for first- and second-year students was 20–24 h per week: 60–90% of sessions were lecture-based with variable amounts of small group, case-based learning (~2 h per week), clinical skills instruction or clinic experiences (~2–4 h per week) and interactive laboratory-based instruction (~0–4 h per week) woven into the curriculum. Lectures were routinely recorded and posted for students as a study aide. At just over a year into the new curriculum, student attendance waned drastically and there were days when only 8 of 82 students were present for lectures. Faculty were discouraged; patients who volunteered to share life experiences with students remarked on the poor attendance. Faculty complaints to administration led to the institution of an attendance policy requiring students to be present for *all* class activities, including lectures. They felt that students’ presence at lectures demonstrated respect and was key to the development of their professionalism. Finally, student attendance was thought to be important for providing fair and accurate teaching evaluations, an important component of faculty portfolios used for promotion decisions.

From 2010–2016, student dissatisfaction rose: students reported anxiety over the lack of flexibility and resentment for being treated like children. Many students physically attended lectures, but were disengaged from the classroom activity. Students began to strategically present themselves for ‘roll call’ and quickly depart once attendance was captured, causing significant disruption to the class learning environment. When we adopted an electronic platform for tracking attendance, students devised mechanisms to message each other so absent students could submit their proof of attendance remotely. This generated student concerns over deteriorating professional behavior and dishonesty among their cohort.

Student complaints drove curricular deans to spend significant time crafting definitions for ‘professional development’ days, ‘grace’ days, criteria for ‘excused absences’ and a formula for how many of each could be utilized. The Associate Dean for Student Affairs was tasked with monitoring/responding to violations reported by a full-time staff member dedicated to tracking attendance. Meetings with students to address absences and the resultant correspondence with course directors about approved absences culminated in approximately 10% of his effort.

Further, and ironically, many students chose to use their ‘grace’ days to skip small group sessions where learning was highly interactive and where engagement was required to achieve learning goals. Requests by students for these allowances were funneled through the Office of Student Affairs, rather than directly through course directors, and did not routinely incorporate faculty into the approval process. Course directors and teaching faculty grew increasingly irritated and felt undermined in their efforts to provide high quality education when students were excused for reasons they suspected they would not have agreed to. This created an environment of growing distrust between faculty and administration about processes to hold students accountable to the policy, often pitting Student Affairs against Curriculum.

In 2015 the UUSOM began to study the value of medical education (defined as [quality + experience] / cost) as part of the American Medical Association Accelerating Change in Medical Education program. We were challenged to measure each component of value for the medical education program, including the effort toward tracking student attendance. The operations to carry out the attendance policy were determined to be very costly with regard to staff and administrator time. Most importantly, in addition to high monetary costs, the policy was negatively impacting the experience of students, administrators and faculty.

Early in 2016, we surveyed teaching faculty about their perceptions of the attendance policy. Although 53% of faculty were in favor of continuing the policy, many of the open-ended comments provided by faculty indicated that mandatory attendance was having negative effects: some themes included “it ‘infantilized’ students and promoted a transactional mentality”, “it eroded trust between faculty and students,” and “negatively impacted the small group learning experiences.” Teaching faculty and students were asked to provide one word or phrase they felt best described the current culture of the school. Stand-out words included *paternalistic, regimented, adversarial* and *entitled*. In June 2016, an education retreat involving senior leadership, teaching faculty, and students was held aiming to realign and reenergize the medical student education culture; as such, the attendance policy and its impact were an important part of the dialogue. At the conclusion of the retreat, participants proposed dismantling the mandatory attendance policy, which was endorsed by the curriculum committee in favor of “encouraged attendance” for all large group learning activities in the pre-clerkship phase of the program beginning with the 2016–2017 academic year. Students were still “expected” to attend large group sessions in which a patient or presenter from outside the university volunteered their time to educate the students, as well as all small-group sessions (such as case-based learning, clinical skills learning community activities, etc.) which comprised ~20–40% of the curriculum.

## Surprising outcomes

We experienced both positive and negative surprises, some of which were anticipated and others which were unexpected when we removed the required attendance policy. Among the positive surprises were that student attendance at case-based learning jumped to nearly 100%. While this was likely attributable to the “expected” attendance policy we initiated for small group sessions, since 2017 90% of first- and second-year medical students have indicated on end-of-course evaluations that case-based learning enhanced their learning. This suggests that students initially attend case-based learning because they are expected to, and continue to attend because they find it beneficial. When the faculty facilitators of these sessions inquired about what policy change had caused such a dramatic rise in attendance, the answer (“We removed the mandatory attendance policy”) typically drew stunned silence. The near 100% attendance at expected attendance activities has remained to the present.

Prior to the removal of the mandatory attendance policy, the school had suffered a several-year decline in both the overall passing rate (93–94% vs. national 95–96%) and mean score (2–4 points below national mean) on the USMLE Step 1 licensing examination. Since the relaxation of attendance expectations, both the passing rates and mean scores have increased to above the national average. In fact, the first cohort of students who matriculated after the policy was dropped had a 100% passing rate with a mean score of 5 points above the national average, and this trend has been sustained (100% passing 2018 and 2019 [national pass rate: 96–97%]; mean: 5–8 points above national). Thus far, providing our students with more responsibility and autonomy for their learning has not hindered their performance on this one high-stakes assessment.

Dissolving the strict attendance policy has removed a significant hurdle preventing a close working relationship between the Student Affairs and the Curriculum teams within Medical Education. While the differing responsibilities between these teams can inherently create good cop-bad cop relationships with students, the required-lecture attendance policy exacerbated these tensions. Not having to make decisions about appropriate reasons for granting excused absences has both eased tensions and provided time for collaboratively identifying goals and processes for an exceptional learning experience.

Although not unexpected, in the years since this policy change, we have seen a decline in in-person attendance at pre-clerkship courses lectures. The UUSOM AAMC Medical School Year Two Questionnaire data reveal that the percentage of students reporting attending pre-clerkship courses/lectures ‘often’ plus ‘most of the time’ has declined from 59% (2016) to 48% (2019), which is still above the national average [8]. While reported attendance has declined, our students’ satisfaction with the quality of their education has risen from 80% in 2016 to 96% in 2019 [8].

We were surprised by a one-year increase in the number of students failing a course during 2018–19, with the common theme provided by the students being lack of engagement with our curricular materials. Additionally, as students have less formal interaction with each other, anecdotal perceptions of students feeling isolated and lonely are increased. The first graduating class to have the attendance policy removed reported a doubling in student-on-student mistreatment compared with the prior class (8.2% vs 16.2%.) [9]. We are currently systematically analyzing these situations to understand the root causes, and are piloting interventions.

Finally, although predictable from prior faculty reactions to poor rates of student attendance, we have needed to actively manage faculty discouragement and expectations. We have provided faculty with analytics showing how many students are accessing materials virtually, even if not in person. We are also helping faculty shift their thinking about what makes a good session: quantity of students or quality of engagement? Instead of mandating a no-lecture policy for our curriculum, we are trying to support faculty in developing the interest and skills in utilizing the most appropriate techniques for the desired learning outcomes. Part of our current conversations are about how to best support students who are disengaging from all non-required attendance activities. We have been pleasantly surprised that many faculty have embraced the call to incorporate more team-based learning and other active learning pedagogies. We hope that the decreased attendance at lecture is only temporary and that when students recognize the increased value of attending in-person sessions, we will see attendance rise again.

## Lessons learned

Akin to what would be considered a medical error in patient care, we believe our process and rationale for deciding to implement an all-in attendance policy in 2010 represented an error in our education decision-making. Had we taken a systems-based approach to analyzing the problem, engaging stakeholders beyond education administrators in identifying and addressing root causes, we likely would have found ourselves in a much different cultural climate as a school. While we originally justified the policy by citing literature linking improved student performance to higher levels of attendance [3, 4], we failed to identify the guiding principles and resultant behaviors that would help us create a student-centered culture.

Our journey from 2010 to now has brought us to consider how important it is to identify the guiding principles we desire as a medical school. Had we taken the time to understand how those principles should have informed our behavior from the beginning, we would have proceeded with our decision-making in a very different way. In 2018, we received additional grant support from the American Medical Association that has allowed us to identify a framework for “education systems thinking.” As a result, we have been engaging students, residents, faculty, and education leaders in defining the guiding principles that shape our work and future goals as a school. Relationships, community, partnership, and student-centeredness are among the core values that are central to our mission as a program. This combined with our effort to focus on the “value” of programmatic changes, we now operate with heightened awareness not just of the monetary costs of the tools, or personnel required to implement changes, but the cost as it relates to the impact on key stakeholder experience. In total, this is helping us re-build a culture of trust and collaboration between administration, faculty and students. Although it has taken years, it feels like a move in the right direction. Now, clear, consistent and non-onerous expectations for student attendance are provided on course syllabi. We trust students to have ownership for their learning and how they spend most of their time. Faculty are critically important to achieving the goals of the education mission; however, their feelings and experiences must not be mistaken for the only and most important considerations. Early on, our focus on the faculty did not afford us the awareness of the downstream consequences our decision would have on student morale and the learning environment. Although the policy drew students to class, they were disengaged and present “only in ‘body’” [10]. Moreover, the policy had an unfavorable impact on the ever-important relationships between faculty, students and administration in medicine. We have heeded Kanter’s [11] advice that “improving the learner-teacher relationship shifts from simply devising ways of getting students to be present, to the challenge of ensuring that both learners and teachers are able to contribute to and benefit from the learner-teacher relationship.” In response, our faculty are asking how to support students who do not attend class, which we believe is symbolic of the beginning of a cultural transformation in which our school is characterized by care [12] and trust rather than paternalism and hierarchy.

## Moral of the story

Instituting required attendance was a faculty-centered, costly, and reactionary decision with unfavorable consequences that impacted the overall culture of our medical school. Owning this mistake has been a growth opportunity for the UUSOM. In 2018 we began a project to define education systems thinking and use it as a framework to ensure that future decisions align with our fundamental principles and our “triple aim” of improving students’ learning experiences, promoting student professional identity, while reducing education costs. Aligning our systems and tools while re-defining our results in accord with the culture we aspire to live and work in has been transformative. We believe schools who take a similar approach will find themselves equally inspired.
